# A Context-Driven Model for the Flat Roofs Construction Process through Sensing Systems, Internet-of-Things and Last Planner System

**DOI:** 10.3390/s17071691

**Published:** 2017-07-22

**Authors:** María Dolores Andújar-Montoya, Diego Marcos-Jorquera, Francisco Manuel García-Botella, Virgilio Gilart-Iglesias

**Affiliations:** 1 Building Sciences and Urbanism Department, Polytechnic University College, University of Alicante, Carretera de San Vicente del Raspeig, s/n, 03690 Alicante, Spain; lola.andujar@ua.es; 2 Department of Computer Science and Technologies, Polytechnic University College, University of Alicante, Carretera de San Vicente del Raspeig, s/n, 03690 Alicante, Spain; dmarcos@dtic.ua.es (D.M.-J.); fmgarcia@dtic.ua.es (F.M.G.-B.)

**Keywords:** building construction process, distributed sensors, smart sensor networks, internet-of-things, last planner system, service oriented architecture

## Abstract

The main causes of building defects are errors in the design and the construction phases. These causes related to construction are mainly due to the general lack of control of construction work and represent approximately 75% of the anomalies. In particular, one of the main causes of such anomalies, which end in building defects, is the lack of control over the physical variables of the work environment during the execution of tasks. Therefore, the high percentage of defects detected in buildings that have the root cause in the construction phase could be avoidable with a more accurate and efficient control of the process. The present work proposes a novel integration model based on information and communications technologies for the automation of both construction work and its management at the execution phase, specifically focused on the flat roof construction process. Roofs represent the second area where more defects are claimed. The proposed model is based on a Web system, supported by a service oriented architecture, for the integral management of tasks through the Last Planner System methodology, but incorporating the management of task restrictions from the physical environment variables by designing specific sensing systems. Likewise, all workers are integrated into the management process by Internet-of-Things solutions that guide them throughout the execution process in a non-intrusive and transparent way.

## 1. Introduction

Technological advances in recent years make it possible to extend the use of Information and Communications Technology (hereinafter ICT) to new applications in order to improve effectiveness and efficiency of different fields and industries. Paradoxically, despite the important role of the building sector in the global economy, the utilization of these technological advances in the construction industry is still quite low. This poor use of technology by construction companies is well known and it has been shown in many studies [1,2,3], emphasizing that the technological reality of the construction industry is far from that of other sectors. 

Another remarkable feature is the heavy dependence of the sector on workmanship, i.e., the use of traditional systems for construction work and its management, which are unstructured and prone to improvisation [4], despite the fact they often involve tasks that could be automated by using advanced technology [5]. This high contribution of manpower—with low specialization [6] and multiculturalism—has been a prominent feature of the construction industry, where foreign workers even represent up to 81.2% of the total construction workforce [7]. This multi-lingual workforce leads to frequent communication problems, due to the language differences [7] and the number of sub-contracting levels arrangement [8], that typically brings together participants from several different firms in the same space. All this becomes a potential barrier for efficient communication and coordination in projects [9], promoting the flow of information to be inaccurate or delayed, and thus, quality failures may occur due to ineffective decision-making [9].

Another relevant problem, related to the heavy dependence on the workforce, the lack of systematic procedures and the lack of technology, is the high amount of rework needed due to errors during the construction phase [9,10], that contributes to the general poor performance of the construction industry [11]. Indeed, up to 30% of construction activities are rework tasks [12], being understood as rework the unnecessary effort of re-doing a process or activity that was initially badly performed, affecting the cost, time and quality of the project [10,13,14]. Specifically, from 6 to 12% of construction costs are wasted on rework of defective components detected late at the construction phase [15,16]. According to [15] 54% of these construction defects are due to unskilled workers or insufficient supervision of construction work. In addition to rework, some defects appear in buildings as a result of errors at the construction phase [17], due to the lack of control of construction work and the physical variables of the work environment during task performance. These causes represent a high percentage of the anomalies seen in the buildings [18], being flat roofs one of the main areas where more defects are claimed [19].

Therefore, the high percentage of defects detected in buildings are related to construction causes that could be avoidable with a more efficient and accurate control through the process automation of both performance and control, and the integration of a systematic information system to improve operational performance and support quality management activities in construction projects.

According to this idea, this paper proposes a new integral management model that includes technology as a fundamental element to improve the performance of building work through an efficient management in the construction phase. This includes the control of the task restriction management at inadequate physical environments. Due to the relevance of the flat roof typology in relation to the high number of defects that shows up, this study focuses specifically on the control of flat roofs during their construction phase. This proposal will enable a greater control of what is built, as well as making better and faster decisions through the integration and availability of all information related to the construction phase, including physical environment information. Also, it will reduce errors and defects, improving quality. To achieve this, it is proposed the use of emerging technology solutions such as smart sensors networks, distributed sensors and Internet of Things (hereinafter IoT’s), using wearables and mobile devices, integrated through a ubiquitous and distributed management system. This system is based on the Last Planner System (hereinafter LPS) [20,21], as a Lean Construction tool for the improvement of the construction workflow [22,23,24,25].

The present work is structured as follows: in the following section we study the state of the art of the research matter. In Section 3, we briefly describe the proposed model and research methodology used during the development of the proposal. In Section 4 we develop an analysis of flat roof construction process together with the identification of defects related to the lack of context information. In Section 5 we propose an acquisition model, focused on the automatic procurement of the physical variables which affect the flat roof execution process. In Section 6 we integrate the acquisition in our general management model focused on the management of tasks and based on an electronic LPS model that integrates construction workers through IoT technologies. In Section 7, we present a prototype implemented together the test performed to validate the system. Finally, in Section 8, the conclusions of this paper are presented.

## 2. Related Works

Smart sensors and data collection technologies have been applied in different areas providing considerable profits by improving real-time information, visibility and traceability [26]. Specifically in the building sector, some sensors and data collection technologies have already been applied for monitoring building process activities. These technologies cover 3D imaging, Global Positioning System (hereinafter GPS), Radio frequency Identification (hereinafter RFID), Ultra Wide Band (UWB), hand-held computers, voice recognition and Wireless Sensor Network (hereinafter WSN) technologies, among others [27].

**Figure 1:**
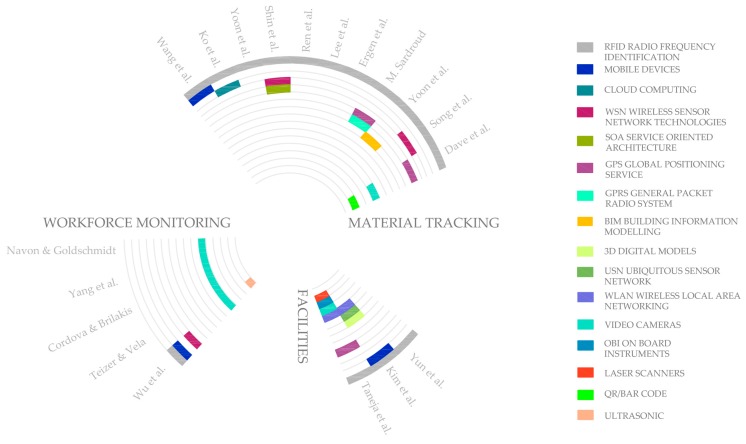
State of the art of smart sensors and data collection technologies applied to the construction of residential buildings.

After an exhaustive review of the scientific literature on the subject, the work presented by Dave et al. [28] that shows the main information streams to be tracked and controlled on site, sensing resources of the execution process through RFID sensors, QR/Bar codes, and GPS, is remarkable. However, after studying other works it has been deduced that most scientific papers that include sensor proposals within the scope of construction are mainly related to supply change management or materials tracking (Figure 1). Accordingly, Wang et al. [29] study the integration between RFID, mobile devices-PDA and websites to get real-time information, facilitate the exchange of information in construction supply chains in Taiwan and provide dynamic operation control. Similarly, Song et al. [30] developed a system that can identify logistic flows and the location of construction materials with better performance using RFID and WSN. Ko et al. [31] also describe the development of a prototype system with RFID and cloud computing in order to avoid recurring problems related to manual management of construction materials, such as lack of materials on site when required, incorrect material supply, and accumulation of material inventories. The same problems on construction projects have also been specified by Yoon et al. [32], who showed the impact of a RFID-based logistics management system on the improvement of managers’ productivity in the specific case of curtain walls. Other authors like Shin et al. [33], also introduce RFID technology, but emphasizing the synergy between RFID, WSN and Service-Oriented Architecture (hereinafter SOA), to collect and share information in supply chain management under the lean Just-In-Time (JIT) delivery approach. Similarly, Ren et al. presented a RFID system to facilitate the management of construction materials in all stages of a water-supply project [34]. Concerning lifecycle management the relationship between materials control and production management was developed by Lee et al. [35] through a framework that includes RFID technology to facilitate the control of materials on construction sites. Furthermore, Ergen et al. [36] determined the benefits of an RFID-based system not only in the construction phase, but also in the whole supply chain including prefabrication phases. Another perspective was applied by Sardroud [37] to automate the identification and tracking of construction materials, components and equipment to provide accurate information to managers with the novelty of the combination of RFID technology with GPS, and General Packet Radio Service (hereinafter GPRS) technology. Finally, also within the area of materials tracking, authors like Yoon et al. [38] integrate RFID technologies with Building Information Modelling (hereinafter BIM) and Motamedi et al. [39] also presented a work linking the usage of BIM and RFID technology, but applied to indoor localization of tagged assets during the operation phase of facilities.

Similarly, other authors use sensors to overcome inefficient material management on construction sites inside the facilities field (Figure 1). Yun et al. [40] determined the reduction of time and cost by using RFID tags in the development of an automated pipeline construction management system. Likewise, Kim et al. [41] presented technologies such as RFID and 3D digital models on a handheld mobile device as an enabling technology for intelligent management of piping installation, including Ubiquitous Sensor Networks (USNs) through Wireless Local-Area Networking (hereinafter WLAN) and RFID to achieve a more efficient task management. Related to facilities, Taneja et al. [42] presented an overview of the different technological solutions of automated data capture in facilities and construction such as image laser scanners and video cameras for image capture; barcodes and RFID tags for automated identification; GPS and WLAN; and On-Board Instruments (OBI).

Furthermore, some of the technologies listed above are used for personnel tracking on construction sites (Figure 1). Video cameras, both statically placed and dynamically moving cameras, were used by Teizer and Vela [43] to monitor the workforce considering four tracking techniques: density mean-shift, Bayesian segmentation, active contours, and graph-cuts. Video cameras for workforce tracking were also presented by Cordova and Brilakis [44] to enable an efficient onsite personnel tracking system. Yang et al. [45] proposed the use of video cameras interacting with the workforce. The same idea was developed by Navon and Goldschmidt [46], who discussed the use of video photography for the location of the workers to improve productivity through an automatic labour performance measurement system. Finally, Wu et al. [47] improved safety performance by proposing an autonomous real-time tracking system on construction sites, through the use of ultrasonic sensors for outdoor and indoor real-time location tracking, RFID for access control and WSN for data transmission.

As a synthesis of the studied literature, Figure 1 shows a graph summarizing the main fields of application within the field of residential building construction. The smart sensors and data collection technologies used in each work studied are also classified and graphed.

Although all technologies specified above improve the efficiency of construction projects from specific perspectives, none of those solutions are focused on improving the construction process itself through the control of internal and external variables. In addition, none of them propose improvements to the core process of residential building construction considering the monitoring of environmental variables, where most quality and efficiency problems originate.

## 3. Proposed Model

Based on the above analysis, a need to propose a novel integration model based on ICTs for the automation of construction work and its management in the flat roof construction process was identified. This model must be able to automatically acquire the context information of the physical environment of the site, and provide it transparently in real time—both proactively and passively—to all the workers involved, guiding the workers during the construction process, indicating whether it is possible to start a task or not, or even if a previously initiated task should be stopped, assisting those responsible for re-planning tasks. Furthermore, it will avoid the future appearance of different defects. In addition, the model provides a traceability mechanism throughout the construction process, to notify if a task has been performed by skipping the system instructions, or even just provide information in the future to facilitate the identification of the root cause of a defect that may appear. To carry out these functionalities, the model must include reliability to ensure at all times the capture of the information and its availability. To do this we must consider energy consumption characteristics and autonomy, data persistence and robustness of the system. Finally, the proposed model must be enough generic and flexible to adapt to the different environments where the execution process will be carried out. Consequently, the proposed model will improve process efficiency, increasing in the same way the quality of the final product, avoiding the costs related to defects derived from the current management processes. Specifically, the proposed model is focused on the flat roof execution process. The main novelties of the proposed model are listed below.

- Identification and characterization of the variables of the physical environment that affect the quality of the construction process during the construction phase.
- Real-time, unattended and automatic access to the variables obtained from the previous process through the use of distributed sensing models and smart sensor networks, an aspect not covered in the field of study so far.
- Design of a novel sensing system suitable for the construction environment.
- Transparent integration of workers and autonomous systems into the process through IoT solutions and integration techniques. This allows guiding workers through the construction tasks from the context information of the worker’s environment.
- Traceability throughout the flat roof construction process.

A research methodology proposed by the research group [48,49,50,51] has been used to carry out the proposal, which is based on business process analysis and modelling for structuring a complex research process into a sequence of tasks, understood as actions that transform inputs into some other output elements. The identified tasks represent a stage of the investigation, and the whole transformation must be aligned with previously identified goals.

**Figure 2:**
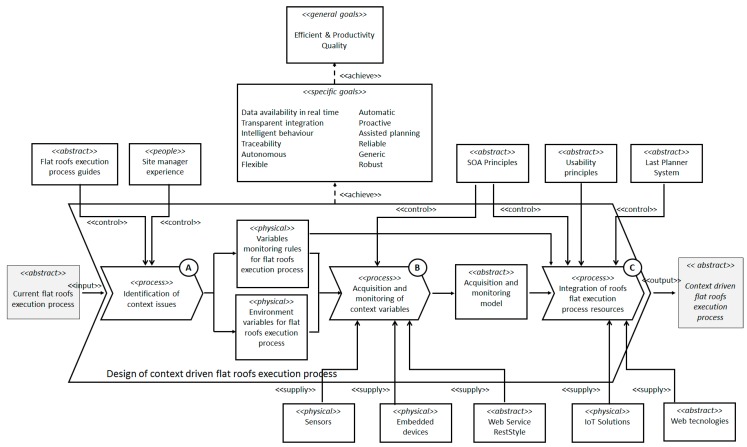
Modelling of the sub processes for achieving automated construction system through Eriksson-Penker notation.

The proposed research methodology is based on Eriksson-Penker formal notation [52]. It is an extension of UML for the representation of business processes that is characterized for being very intuitive and easily understood by all the stakeholders involved in the process. This is a notation that facilitates the understanding of the problem and its subsequent analysis to identify a solution according to the objectives. This notation was chosen because, unlike others business process graphical representations, such as the Business Process Model Notation standard (BPMN), includes a set of graphical artifacts and relationships (such as controllers, suppliers, goals, input and outputs) whose specific semantic was more suitable to achieve our aims. These artifacts allow us to represent the different techniques, paradigms and technologies used in each stage of research through transformation processes. This is embodied in Figure 2 where the starting hypothesis shows the main process carried out in the research, where the input element (<<*input*>>) represents *current flat roofs execution process* in traditional construction. This <<*input*>> must be transformed through this design process into a *context driven flat roofs execution process* (<<*output*>>), meeting the requirements identified above and which now represent the strategic objectives that will guide the research process (<<*specific goals*>>).

To achieve these strategic objectives (<<*specific goals*>>) represented in Figure 2, the controllers (<<*control*>>) and facilitators (<<*supply*>>) must be established to guide the transformation. Those represent strategies, paradigms, techniques and technologies that will be integrated into our proposal. The defined process will achieve the object model of this work in a systematic way, selecting the most appropriate techniques and tools to meet the objectives and thus solving the problems identified. In the proposed methodology, each of the tasks identified represents a stage of the investigation, and these will have associated one or more scientific methods as described below.

The transformation process that conducts the research has been divided into three phases or stages which are shown as three sub processes. The first sub process is denominated *Identification of context issues* (A in Figure 2). The objective of this phase of the research was to identify the variables of the context and the physical environments that affect the quality of the roof building process, being that—as it is specified in Section 4—the main cause of the habitual defects. Moreover, at this first step of the research are identified the recommendations and best practices that should be followed, for the measurement and incorporation of such information during the execution of the process.

The second of the sub processes named *Acquisition and monitoring of context variables* (B in Figure 2), aims to design an intelligent system for the acquisition and automatic monitoring of the context information identified in the previous sub process. In addition, the system must provide such enriched information giving an added value in the roof execution process, in order to help decision making during its execution.

**Figure 3:**
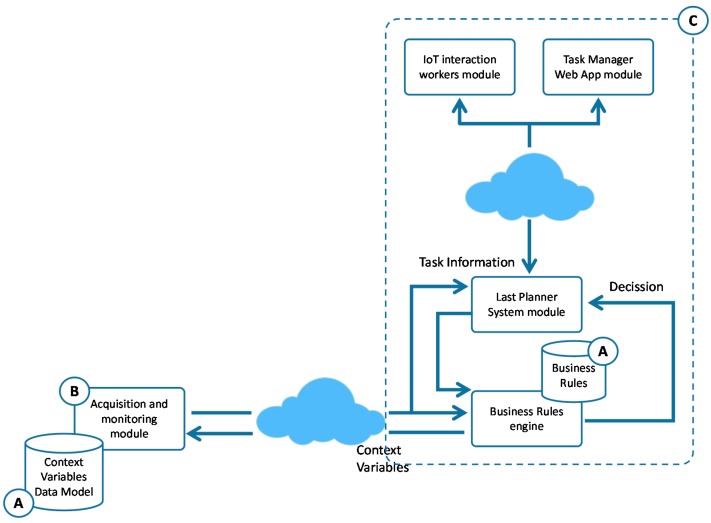
General architecture of the proposed model.

Finally, the third sub process named *Integration of flat roofs execution process resources* (C in Figure 2), aims to integrate all the actors and resources involved in the process to provide an assisted system for the execution of the process, and therefore, to avoid the current bad practices that origin defects. At this point information, technologies and human resources must be integrated in the process in a transparent way to achieve the previously stated objectives. As a result of all phases carried out at each stage of the research process, it is presented below the general architecture of the proposed model (Figure 3) which will be described in detail in the following sections.

## 4. Identification and Analysis of Defects

The main sources of building anomalies are design errors and construction problems [17], partly due to the lack of control of construction work. These causes represent around 75% of the anomalies, compared to 22% due to lack of maintenance and 3% owing to accidental phenomena [18]. Therefore, a high percentage of defects detected in buildings associated with construction causes could be avoidable with a more efficient and accurate control of the process. According to the MUSAAT Insurance Foundation [19], roofs represent the second area where more defects are claimed, about 17%. Specifically, anomalies in roofs come from a simultaneous contribution of several variables, 75.92% associated with flat roofs while 24.08% is associated with pitched or inclined roofs [19]. Due to the relevance of the first typology of roofs, this study focuses specifically on the control of flat roof construction during its execution. In this context of anomalies in flat roofs, most of the defects have their origin in the waterproofing process [19]. The same assertion is repeated in the literature where some authors like Walter et al. [53] conclude that the majority of the cases of defects found in flat roof have their origin in the waterproofing phase, mainly due to application errors and environmental actions. Accordingly, most of them could be reduced or even removed with a correct performance and control during their execution. Within the frame of operational errors, they include the following aspects as possible causes of failure, among others: personnel inexperience, bad execution of the gluing, bad execution of the welding, application in humid/rainy weather, bad smoothing of the finished surfaces, deficient cleaning of the support, inexistent/deficient supervision/quality control, deficient waterproofing positioning, overly short due time, insufficient material quantity, etc. Likewise they list the following environmental actions as variables that affect the execution of roofs generating future defects, these environmental actions referred to are wind, heat, ultraviolet radiation, humidity, natural ageing, humid—dry cycles, etc. [53]. Although the greatest numbers of defects in flat roofs are identified as dampness by filtration, there are other defects that affect the roofs to a lesser extent but they are equally considered relevant in the present study (Table 1).

Among the possible causes of failure related to previous defects are the lack of control in the singular points of the roof, as well as deficiencies in slopes [19] that must be between 1% and 5% according to Spanish regulations or lack of flatness on the support surface [54]. For the same reason the base for the waterproofing must be clean, uniform, dry and without dust before starting the next task. In addition, work should be suspended under atmospheric conditions like rain, strong winds, snow or when temperatures are very high. Moreover, other deficiencies like dampness appears on the underside of the support when the water on the slope formation layer is not allowed to dry because this is hindered by the placement of other materials. Likewise, the prolonged exposure to the weather of some materials like waterproofing membrane during the execution phase—under too high or too low temperatures—produces embrittlement in the material also causing the appearance of dampness [54]. Other common deficiencies such as humidity caused by filtrations are related to the protective layer, which are usually generated by poor design and an improper execution on site [55]. Specifically for walkable roofs with exterior floors—according to the recommendations of use of the Spanish Association of Manufacturers of Ceramic Tiles, Paving and Tiles [56]—in order to avoid bulging in the tiling, lack of adherence in tile parts and tiled cracking the surface that will receive the gripping material to install the tiles must be completely clean before beginning the installation. Otherwise unremoved waste will generate weak bonding points that can be the origin of the above defects and future detachment of floor parts. It is also necessary to have a perfectly dry surface (always below 3% humidity), flat (less than 3 mm deviation in 2 m in any direction). Likewise, the optimal environmental conditions are temperatures between 5 °C and 30 °C, when there is no rain or excessive humidity, avoiding the risk of frost, wind and strong air gusts. Besides that, the surface must not be wetted within 48 h after the exterior floor placement [56].

**Figure 4:**
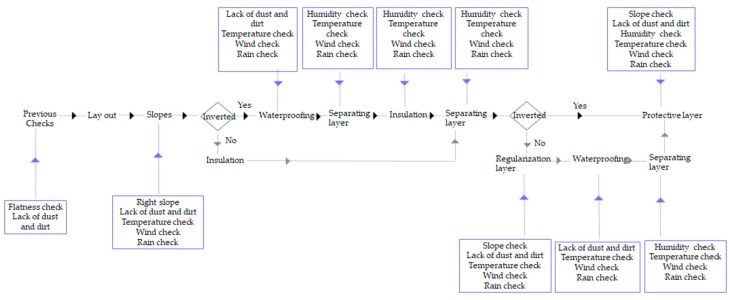
Variables that cause most of the defects in walkable flat roofs.

Accordingly, Figure 4 shows the main variables that affect the quality of the work during the construction process of walkable flat roofs whether they are traditional or inverted, i.e., the environment conditions together with the main essential requirements, for proper performance of activities to prevent future occurrence of deficiencies. Some of these environmental variables relate to adverse weather conditions or just weather conditions incompatible with the works to be performed. These weather variables are rain, very high temperatures with excessive sun exposure, or very low temperatures below 5 °C that produce an excessive delay in the setting of mortars and concretes and therefore poor development of resistance, excess humidity, bursts of wind, etc. Nevertheless, work variables are related to the human factor, i.e., poor performance of activities and lack of supervisions that prevent compliance with the recommendations of Spanish Association of Manufacturers of Ceramic Tiles.

According to this, it is possible to classify the variables (Table 2) regarding whether the control of these variables—and the verification that they are within their acceptable limits—is something specific (S in Table 2), i.e., when the control is realized in specific moments, or continuous variables (C in Table 2) whose control must be done with a certain frequency. In continuous variables, it is usually of interest to contemplate their temporal evolution as a criterion in decision making during the construction process, such as the evolution of temperature or humidity throughout the day. From the variables identified in the Figure 4, Table 3 shows the limits that previous conditions must comply with, as well as the list of sensors proposed in the present work to achieve a total control over the execution of the walkable flat roofs.

## 5. Acquisition and Monitoring of Context Variables

**Figure 5:**
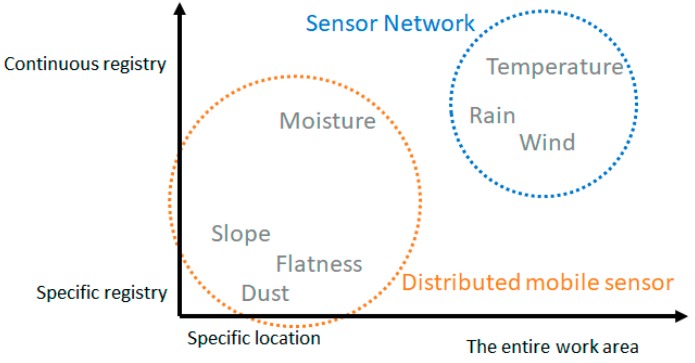
Classification of variables by extension and temporal distribution.

Context variables related to the construction process of a flat walkable roof were identified in the previously performed analysis. These variables are of a different nature and can be classified according to their acquisition process. According to the extension that variable affects, we can classify them as specific variables that affect a specific area of the work surface, or as general variables affecting the entire work surface. Then, Figure 5 shows all variables previously analysed, classified by extension and temporal distribution.

As shown in the Figure 5, two distinct groups of variables can be identified. For those that require the collection of information on more or less extensive surfaces, we propose the use of sensor networks. Sensor networks allow covering large areas easily since they use an ad-hoc network that does not require support infrastructure, they are flexible, easy to deploy and self-configurable [57]. This makes sensor networks a suitable proposal for variable and poorly controlled environments, such as this field. Each node of the sensor network works in coordination with other nodes, it contains acquisition modules required to monitor the variables defined, and it has enough wireless communication capability to transmit the captured information.

In addition, to achieve the proposed objectives, each node will have the ability to store and process information in order to provide persistence, reliability, and fault tolerance; as well as to standardize the captured information. For the subsequent access to the monitored information, the network will have a Gateway that will allow access to the network from the Internet. In addition, we propose the use of distributed mobile sensors for the variables that require data collection at specific points and at specific moments. These sensors have as main feature their own mobility, allowing an operator to move them at any time anywhere on the roof where a punctual capture can be required.

**Figure 6:**
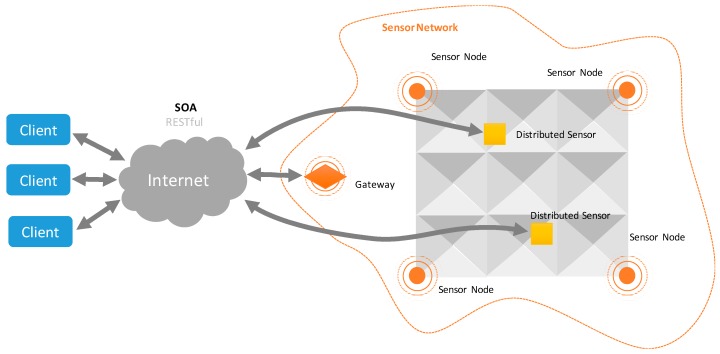
Distribution of sensors on a roof.

For example, an operator may position the movable sensor on the surface of the roof to check the soil moisture at a point or its inclination. Unlike sensor networks, these distributed sensors work independently and do not require other sensors to perform their function. Like every node in the sensor network, any distributed sensor has various capture modules, as well as communication, storage, and processing ability. A general outline of the distribution of sensors on a roof is shown in Figure 6.

One of the main differences between the nodes of the sensor network and the distributed sensors is their energy management. The sensor network nodes, since they are highly flexible and can be distributed in multiple ways, cannot always have a sufficient source of energy, and therefore, they have to make use of batteries or alternative power generation systems, so to achieve a high autonomy, its consumption has to be limited. However, in the case of our distributed sensors—a mobile sensor with specific use—autonomy is not so critical, since batteries can be charged periodically.

**Figure 7:**
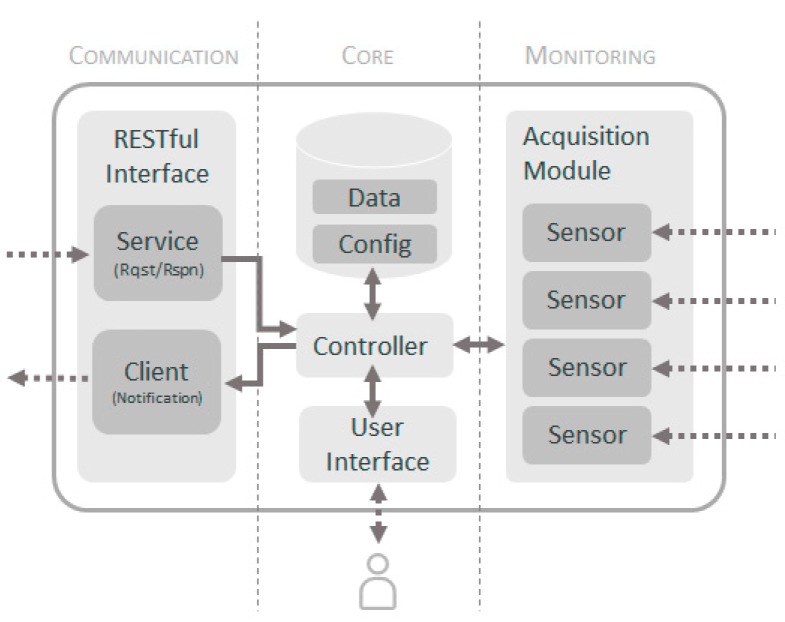
Architectural model of the sensors.

Figure 7 shows the architectural model of the sensors. In this model three clearly differentiated parts are distinguished. A monitoring block composed of an *Acquisition Module* that coordinates the monitoring of all variables. This module integrates the various sensing modules that perform specific acquisition tasks for each variable (temperature, humidity, wind, etc.). The system can incorporate as many sensors as variables need to be monitored, so that the proposed objectives of flexibility and generality can be achieved. Note that the Gateway of the sensor network will not have an *Acquisition Module*.

The core of the sensor will contain an information system, where both the values of the monitored variables and the configuration of the sensor will be stored, where it will be indicated which variables must be monitored, how often and with what parameters. To coordinate the entire monitoring process, we propose to use a *Controller Module* that will centralize all the logic of the sensor. Finally, there is a *User Interface Module* that allows the interaction of a user with the sensor through peripherals (pushbuttons, screens, etc.). Then, the model is completed with a communication area based on REST style Web Services to provide the information to other elements of the system. This module is only present in distributed sensors and in the Gateway of the sensor network.

### 5.1. Sensors as a Service

**Figure 8:**
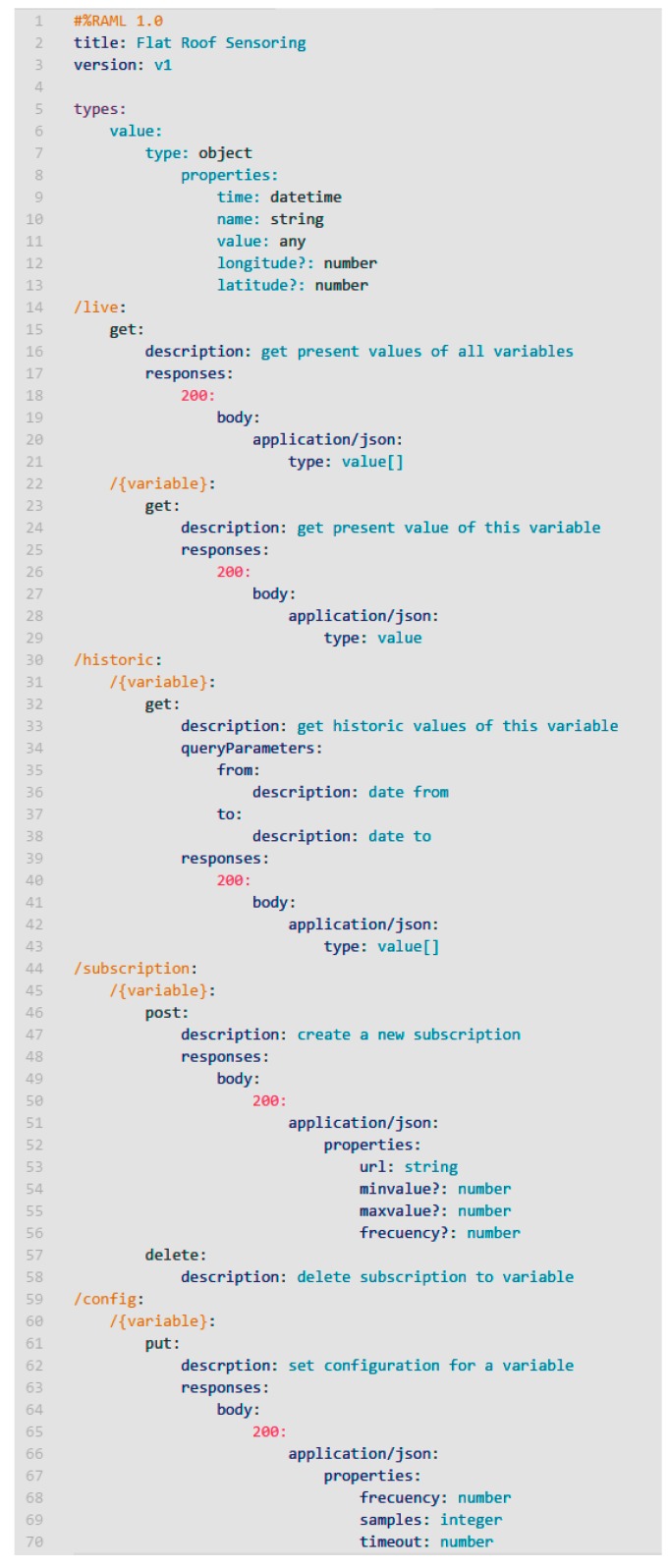
Monitoring flat roof service defined with RAML.

To achieve a high level of integration of the monitoring system with the rest of the system elements, we propose the use of SOA as integration paradigm. The use of SOA contributes fundamental features to the model to achieve the proposed objectives such as interoperability, low coupling, standardized access to information, reusability, composition, and scalability. Specifically, in the present research, REST style Web Services were used, achieving a slightly more decoupled and a light communication model much more suitable for these environments. Through REST services, both distributed sensors and gateways of sensor networks, it is provided a standardized API that allows external consumers to remotely access the monitored variables, as well as configure the settings that the monitoring system includes. A summary of the API defined with RESTful API Modelling Language (RAML) it is shown in Figure 8, where the main endpoints and responses of the web services are reflected.

Access to the monitored information may be carried out in two possible ways. On the one hand, you can explicitly request the necessary variables using a request-response pattern, where the sensor acts as a passive element of the system, providing the requested information. It can be consulted the current value of the variables (path/*live*), as well as the historical value of stored values (path/*historic*) between certain dates. Another access mode use a notification pattern, where initially an external agent subscribes to a certain variable (path/*subscription*), and the sensor actively sends information about the variable; either when it changes its value, or when the value changes from a defined range, for example, exceeding a threshold.

The notification is also made by RESTful service to the URL specified in the subscription. This mode allows the capture system to behave proactively, informing other parts of the system about states of context that must be taken into account when performing certain tasks, for example warnings by temperature, humidity or very high wind.

In addition, the use of API service makes possible the configuration of sensors (path/*config*), establishing for each variable the sampling frequency, the number of samples to be taken and mediate each shot (in order to minimize capture errors), and the maximum timeout of the sensor to report that it has not been able to perform a certain reading. This parameterization of the sensors has a qualitative effect on the flexibility and performance of the proposal.

## 6. Integration of Flat Roofs Execution Process Resources

One of the most novel aspects of the proposed model is the way in which the context information of the physical environment—is included in the management during the construction process of flat walkable roofs. This aspect transparently assists workers and managers in their tasks. The objective is to make this information accessible and available anytime and anywhere. Thanks to this, involved workers know if they can carry out the tasks previously planned and assigned, or if instead it is necessary to perform task correction in planning. In this way, it will be possible to avoid the appearance of future defects and provide a more efficient work method to ensure quality.

**Figure 9:**
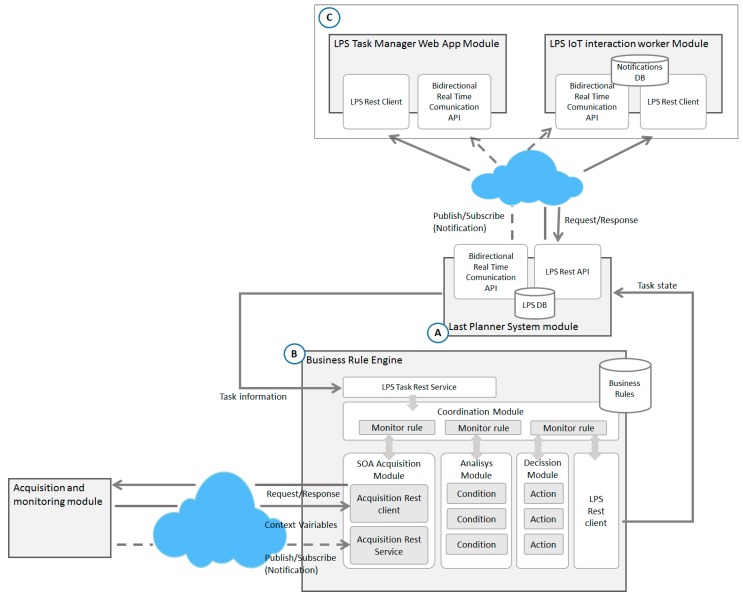
Architecture for LPS module, Business Rule Engine and interaction modules.

Another aspect of the proposed model to consider is that ensures traceability of all relevant information of the work in the LPS DB (A in Figure 9). It allows, in case of anomalies or defects, to identify the cause in the future through the analysis of such information. To achieve all these objectives, different methodologies, techniques and technologies such as LPS, IoT solutions, Usability principles, SOA and Web technologies have been used in this integration phase. As the main element for the integrated management, the model is based on a module of management and control of tasks based on LPS. This is a Lean tool for the management of the construction processes which improves efficiency and reduces the uncertainty derived from the process. LPS has several phases to achieve its purpose, including an initial phase of overall planning of the construction project (Master Scheduler); a phase of medium-term planning to avoid problems related to constraints and dependencies between tasks (Look Ahead) and a short-term planning phase for weekly management and control (Weekly Work Plan).

Due to the variability of the context information from the physical environment, it must be considered in a short period of time to ensure accuracy. For this reason, the information acquired by the acquisition module will be integrated into the LPS phase called the Weekly Work Plan. The Weekly Work Plan phase reflects all tasks of the detailed weekly work plan based on reliable commitments. As in the other phases of LPS this information is shown through stickies, stuck on a wall located in a specific physical space prepared for it, representing the weekly planning. Thus, the participants can follow the evolution of the tasks in which they are involved. To achieve the objectives, the proposal is based on three main actions.

First, the model has been based on the proposal of a digital LPS system (A in Figure 9), implemented through a friendly interface based on Web technologies. The interface provides a metaphor for “Kanvas” and “post-it” that includes a panel where with a simple process of “drag and drop” activities and tasks for each week are easily distributed. Besides, it allows the management of their states and incidents that may occur, as well as the allocation of resources associated with each task. Through this proposal, the information of the planning and execution of the tasks will be accessible by all workers involved from any site and device.

Secondly, Weekly Work Plan reflects the reliability of being able to start tasks depending on resource constraints, but it does not take into account the constraints derived from causes associated with the physical context conditions in which they are carried out. The present proposal includes the necessary functionality in the LPS to incorporate such information in the process. Then, it has been defined a business rules engine responsible for determining the feasibility of tasks execution, connecting the LPS module and the sensor system. The *Business Rules Engine* is composed of a set of functional elements that are detailed below (B in Figure 9).

- The business rules information system stores the configuration information of rules whose structure is based on the analysis performed in Section 4 (see Table 2) and are defined through the following tuple:
BR ≡ (Task, Var, Conf, Cond, Act)
(1)
  where *Task* represents one of the tasks planned in LPS for which context information must be verified to determine whether it can be executed or not; *Var* represents the set of physical variables of the environment which must be acquired and analysed to determine if the task should be executed or not. These variables are provided by the previously designed sensing system; *Conf* represents the monitoring configuration to obtain each of the variables mentioned above. It determines the pattern of acquisition of the variable (request-response or notification), the endpoint of the sensor service that provides the variable and, if applicable, the frequency of acquisition; *Cond* represents the conditions that must be evaluated from the variables to determine if it is possible to perform the action or not. These conditions can be determined through simple, multiple comparisons, formulas or script functions. The result of the conditions will be a Boolean value that indicates the possibility of executing the task or not; *Act* represents the action that must be carried out once a condition is evaluated.

Such configuration must be performed before the beginning of the construction project.

- *LPS Task RESTful Service* is the input service to the rules engine that exposes the functionality for receiving tasks that must be verified before execution and these are sent from the *WWP module*. That communication comes just in time prior to the beginning of a task, if it is required to work with a reactive model. However, if a preventive mode is required, then, the communication is made in an instant of time determined by the manager. That instant consists of a period prior to the planned for the execution of the task.
- *Coordination Module* is responsible for managing the life cycle of different monitoring agents. For each validation request that arrives at the *LPS Task RESTful Service*, the *Coordination Module*, from the configuration for the specific task, launches a *Monitor Rule Agent*, which is responsible for coordinating the entire flow of the verification process during the life cycle of a task and encapsulates the behavioural flow of the rule.
- The *SOA Acquisition Module* is the module responsible for obtaining the context information, through the sensors, related to the task to be validated and sent to the corresponding *Monitoring Agent*. This information will be obtained in two ways: (1) Through the *RESTful Client Acquisition Component* through a request-response pattern if it is a case of isolated acquisition variables and on-demand; (2) Through the *RESTful Service Acquisition Component*, that implements a publication-subscription pattern towards the sensor services and receives notifications when a continuous monitoring variable changes.
- The *Analysis Module* is the module responsible for determining, from the context information, if a task can be executed or not. This functionality is performed through the *Condition Component* associated to each task to be evaluated. This component receives the required context information through the corresponding *Monitor Agent*, applying the conditions for which it was configured. As a result, it returns a Boolean indicating the adequacy of the execution state of the task.
- The *Decision Module* is responsible for determining the status of the task through the *Action Component* associated with each task. This component receives the analysis result through the *Monitor Rule Agent* and returns the updated status of the task (continue, stop, risk).
- The *LPS RESTful Client Component* is responsible for transmitting the resulting state returned by the *Action Component* to the *LPS Module*, which updates the information and is responsible for communicating it to the actors involved as we will see below.

Finally, information to guide the workers involved is necessary, all this through a proposal that makes the complexity of the system transparent and with barely intrusive characteristics in the daily reality of workers. For this, the model has been based on two modules of interaction (C in Figure 9).

The first, through a usable interface designed by Web technologies, which is oriented to the managers of the execution process. Thanks to this, they can have a global view of the process in real time and from anywhere, in addition to the restrictions on planned tasks by the context information of the physical environment and the actions of all workers. The second, oriented to workers so that they know at all times the management of specific tasks in which they are involved, it is based on IoT solutions such as the inclusion of a SmartWatch or SmartPhone, elements of daily use in most of the population. The module will offer a very basic and usable interface in order to avoid technological rejection with the following functionalities.

- Integrate workers in the process with IoT solutions and basic functionalities through a usable interface.
- Identification of the worker. Any worker will be identified against the server before starting their tasks. Then at this point subscription is automatically made so that the *LPS Module* notifies the worker of all the information about the tasks in which it is involved. Workers are identified through user name and password. The server validates credentials and returns a token to identify all their requests. In addition, for the asynchronous communication, once user is authenticated, the server stablishes one communication channel associated with user’s device.
- Notifications. The *LPS Module* will report in real time any situation that affects the tasks of a specific worker, from the tasks to be done, incidents occurred or even task status changes due to information from the physical environment. Each time a notification occurs, the system will use an audible alarm and a vibration mode so that the operator is aware of the information.
- Management of tasks and incidents. Each worker can communicate to the *LPS Module* the current situation of its work in relation to the assigned tasks. If the worker starts the task, if pauses, if finishes, or if there is an incident that should be reflected, etc.

## 7. Implementation, Testing and Validation

In order to validate the model, a prototype of the system was developed to assess the adequacy of the proposal to the objectives set at the beginning of the investigation. In this chapter, both a test scenario and a set of experiments were designed to validate the proposal in a formal and systematic way.

### 7.1. Prototype Design

This section describes the design and implementation of the prototype based on the proposed model. The complete prototype consists of parts defined in the general architecture: an acquisition and monitoring system, an LPS system and a business rules module. The prototype also includes a set of interfaces that allow the integration of users into the system.

#### 7.1.1. Acquisition System Prototype

Due to the constraints imposed by the actual environment in which the capture system will operate, where a stable power outlet is not guaranteed at all times, it was decided to use as base an embedded system of low power consumption by batteries. In particular, a Raspberry PI platform that not only meets the requirements, but also has a wide range of sensor modules was used. Table 4 lists all the hardware elements used for the prototype, for both the distributed sensors (DS), the nodes (NSN) and the Gateway (GSN) of the sensor network.

For both DS and GSN prototypes, since they have to communicate with external elements the third version of Raspberry Pi was used because, although its power consumption is slightly higher, it has a built-in WiFi module. In any case, although we did not use it in the prototype, it would be also possible to incorporate a 3G/GPRS communication module for environments where there is no WiFi coverage.

For the configuration of the sensor network, the 802.15.4 standard, using Xbee modules was chosen, which provides a reliable network with a range of up to one mile between nodes.

Although the devices are autonomous, and their fundamental work is done and configured remotely, a lightweight human-machine interface was added to the device to allow in person consultations and basic configurations. For the user interface, and due to space and consumption constraints, both an LCD display and a potentiometer with rotary encoder are used for displaying and entering data.

In order to power the device, in the case of DS a battery was chosen, since it has punctual use, and it can be easily changed to charging by cable. In the case of nodes in the sensor network, since their position is more static (typically fixed on certain parts of a roof), a battery with a solar panel was chosen allowing its charging during daylight hours, which significantly improves their autonomy.

**Figure 10:**
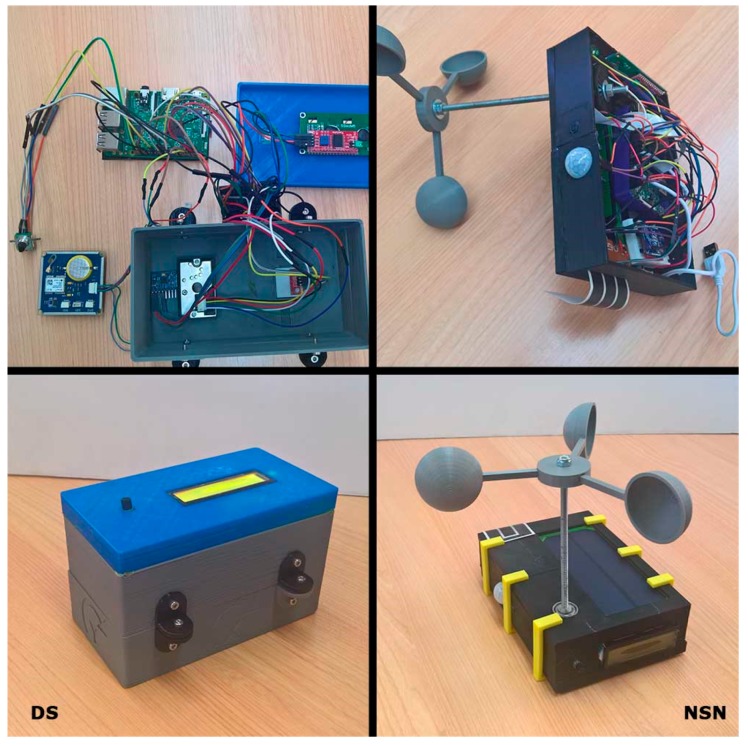
Picture of the implemented sensing system prototype.

Since all the devices will be used outdoors, a hermetic encapsulation was made to protect them from the sun, rain and humidity. This encapsulation was custom designed and printed using a 3D printer. The wind turbines were also printed in 3D, and together with a photointerruptor for the count of steps, make up a wind sensor. Moreover, a sampling is performed for 5 s to determine the speed, and the number of steps is transformed into velocity by a factor determined by a previous calibration. Figure 10 shows the final results of the prototypes for DS and NSN.

**Figure 11:**
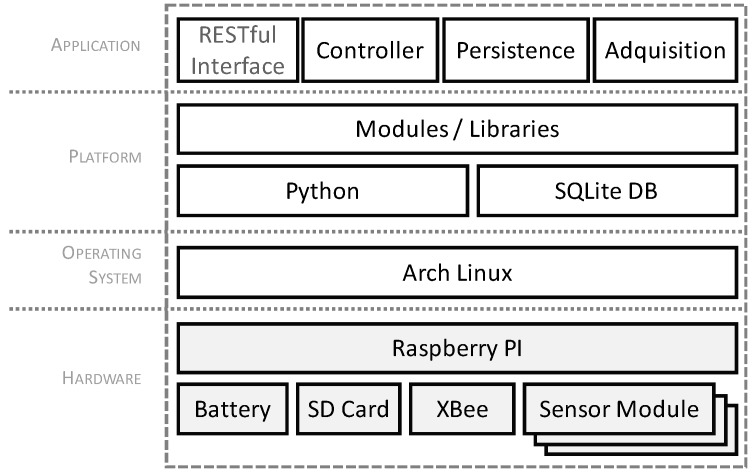
Layered architecture of the prototype.

For the software development of the device, an *ArchLinux ARM* distribution compatible with Raspberry PI, and with a development platform based on Python has been chosen. Figure 11 shows the layered architecture that was used in the implementation of the prototype. As a storage system, a SQLite database was used, since it perfectly meets the requirements of the developed application. Furthermore, its lightness makes it suitable for an embedded platform.

In Table 5 we can see a list of the software components used for the prototype, and their versions. The modules of the application layer, which correspond to those defined in the model of sensor proposed in Figure 11, were implemented in Python by using various programming libraries. For the RESTful interface, the Flask framework was used for services, and the Request library for service invocation in active mode.

#### 7.1.2. LPS Prototype

For the development of the prototype of an LPS system, a design based on Web Technology was chosen. This allows us to achieve the goals of ubiquitous access, enabling anytime, anywhere access to updated information about construction work.

**Figure 12:**
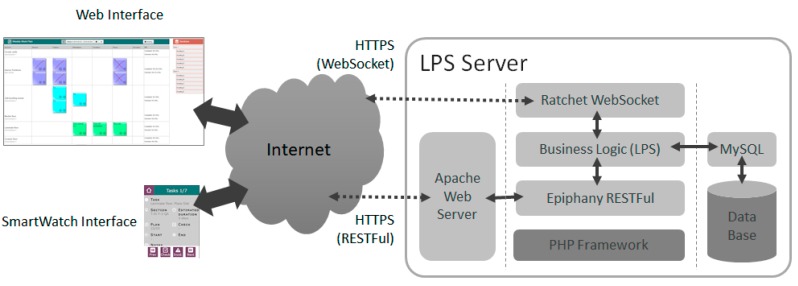
Prototype LPS.

It was decided to use REST style Web Services, which has allowed its easy integration with the acquisition system. Authentication was achieved through JSON Web Token (JWT). The development has been done using PHP, using the Epiphany library for the implementation of web services, Apache web server as HTTP server and MySQL as DBMS (Figure 12). Each entity in the LPS DB (activity, task, restriction, …) was implemented as a RESTful service, to be consumed by different clients. Additionally, so that synchronization between all elements that access LPS is instantaneous, and the server is not saturated through an access based on pooling models, a channel based on the technology Web Socket was incorporated to allow a bidirectional communication with the server. In this channel, it has been implemented a notification system with which the server can notify the connected elements that a change or an event has occurred in the system. Its implementation has been made through the Ratchet library.

As interfaces for LPS a web using the AngularJS v1 library, and an application for the Samsung Gear smartwatch developed in Tizen were developed. Both interfaces access the LSP consuming the implanted web services and subscribe the system notifications through a Web Socket. As shown in Figure 12, security features were achieved by use of a secure channel layer based on HTTPS.

### 7.2. Test Scenarios

**Figure 13:**
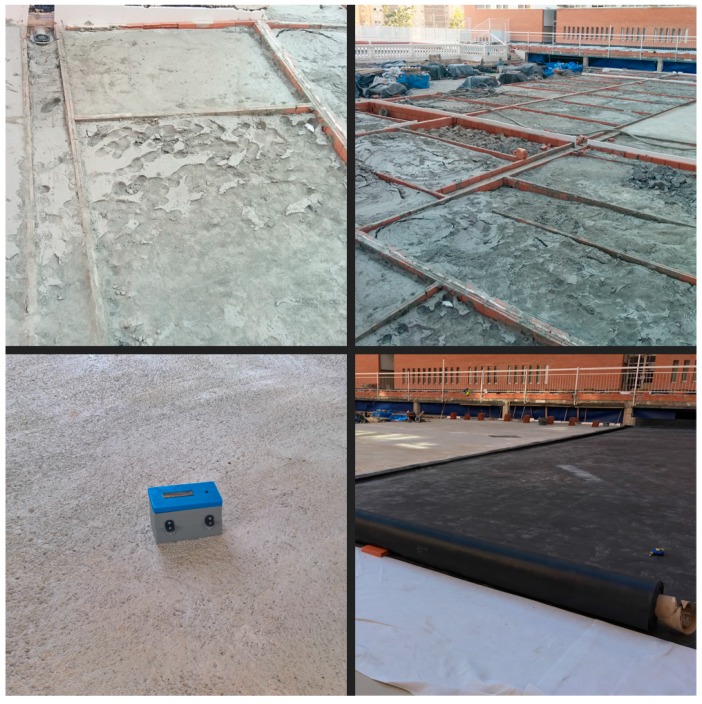
Test scenario in a real environment.

Two test scenarios destinations were designed to validate the research. One of them was developed in a laboratory to validate the system in a controlled environment, and to measure the performance of the prototypes. In addition, stress tests were performed to know the maximum limits and capacities of the proposal. Another scenario (Figure 13) was designed in a realistic environment, setting the prototype in motion in a real environment during a flat roof construction process. These tests validated the proposal in a realistic environment. Specifically, the tests were carried out during the construction of a residential tower of apartments. The work was performed by a subcontracted company (four workers) in Alicante (south-east of Spain) in February 2017. Next, it is described the set of experiments that have been performed, indicating for each of them both the objectives and the results obtained and the conclusions drawn. 

#### 7.2.1. Experiment 1: Performance of the Capture System

The first experiment aimed to validate the capture process of the different variables supported by the capture system. For this purpose, 500 read requests have been made for each variable of the capture system. The overall time of the request-response process has been measured, including the processing times of the REST service, the time spent for the sensor to obtain the value, and, for the case of the sensor network, the time spent transmitting the data from the sensor node to the Gateway.

**Figure 14:**
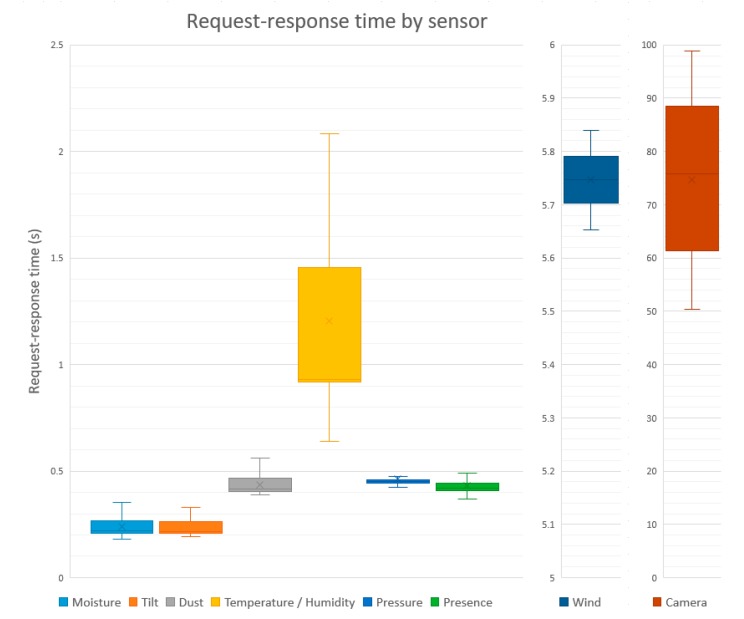
Box plot of Request-Response time results.

The results are shown in the Figure 14. These are box plot graphs, which provide information about the dispersion and asymmetry of the data. As shown, the response times of the sensor network depend on each sensor. Moisture, tilt, dust, pressure, and presence variables take less than 0.5 s. Temperature and humidity sensor is slightly slower than the rest and is delayed more than a second. In the case of the wind sensor, the high value is given by the fact that a sampling is carried out for 5 s. The sensor that takes the longest time is the image acquisition, due to the high cost of transmitting an image of several hundred kilobytes using the XBee network. In any case, the access to the set of variables is quite fast and can be considered adequate for the objectives of the research work.

#### 7.2.2. Experiment 2: Consumption and Autonomy of the Capture System

The second experiment aimed to validate the autonomy of the devices. Power consumption samples were recorded in the three prototypes, differentiating them into four different states. The first sample was in a standby state, with no sensors connected and no data processed. In this case the best consumption was given by the NSN, since it used version 1 of Raspberry Pi, compared to the DS and GSN that have version 3. For the second sample, all the peripherals were connected to the prototypes. In this case the biggest jump in consumption occurs in the DS, mainly due to the use of sensors such as GPS. Subsequently, for the third sample, the monitoring system was started up, which initializes certain sensors, being the greatest of the increments the one suffered by the NSN.

**Figure 15:**
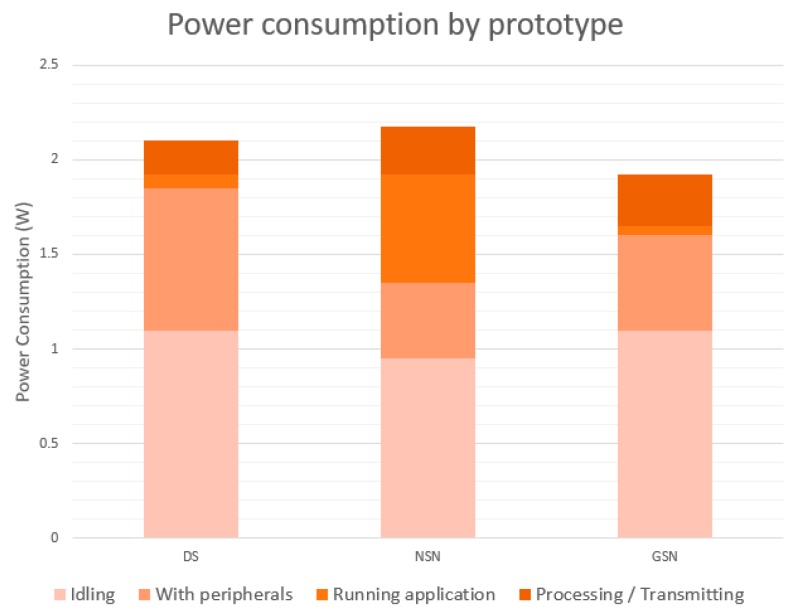
Power consumption by prototype.

Finally, a fourth measure was taken with the device at maximum performance, using the communication processes to transfer the information. As a final result (Figure 15), it was observed that all the prototypes had an acceptable consumption, close to 2 Watts, making it a suitable solution for the proposal.

The autonomy of the devices will be determined by the actual use given to each of them. The DS, when acting as a specific sensor, turns on when sample is to be taken and subsequently (after 5 min without being used) automatically turns off. For this test, it has been kept on steadily and its duration has been 16 h and 30 m. In the case of NSN, the maximum continuous duration using the panel on a sunny day was 26 h and 11 min. In order to improve the autonomy of these devices, a power management module was used to allow the planning of switching the device on and off for the specific moments in which the shots will be made.

#### 7.2.3. Experiment 3: Acquisition Service Behaviour

The objective of this experiment was to validate the adequacy of the sensor network as a service. For this purpose, several request sequences have been made reading the values of stored variables. The aim was to obtain the behaviour of the REST services of the sensor prototypes regardless the capture process of information previously validated.

**Figure 16:**
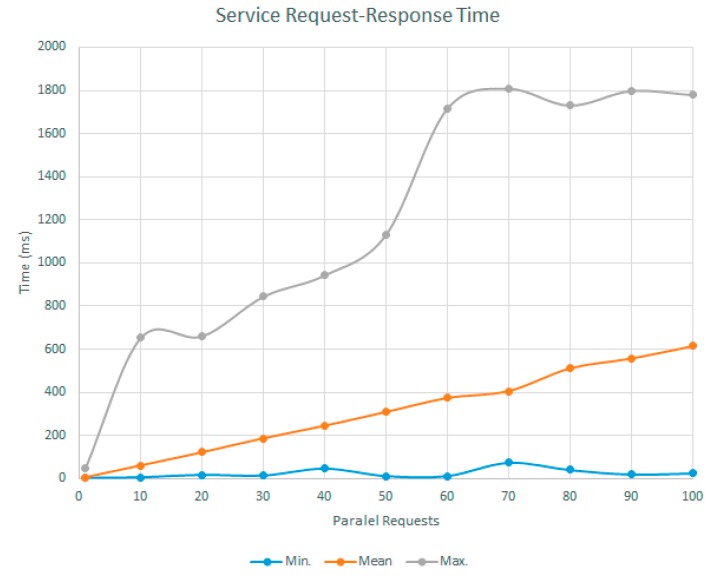
Request-response time of the service implemented in the prototype.

Each series of requests consisted of 10,000 requests, and in each series the number of requests that were made in parallel was increased. The result can be seen in Figure 16, where it is observed that the mean response time although the number of parallel requests increased, it remained quite stable and within adequate ranges for this research work. In the case of the maximum response time collected by each series, the values were also appropriate for the proposal.

#### 7.2.4*.* Experiment 4: Integration

**Figure 17:**
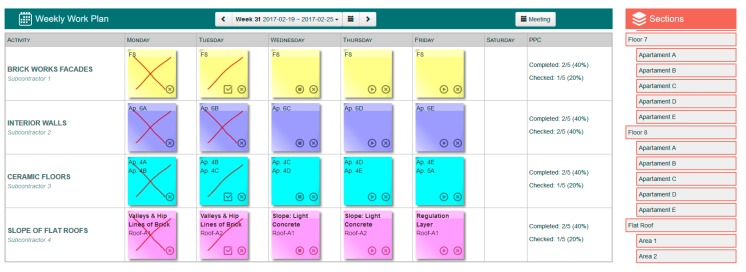
Screenshot from the LPS WebApp of the weekly work plan at week 31.

**Figure 18:**
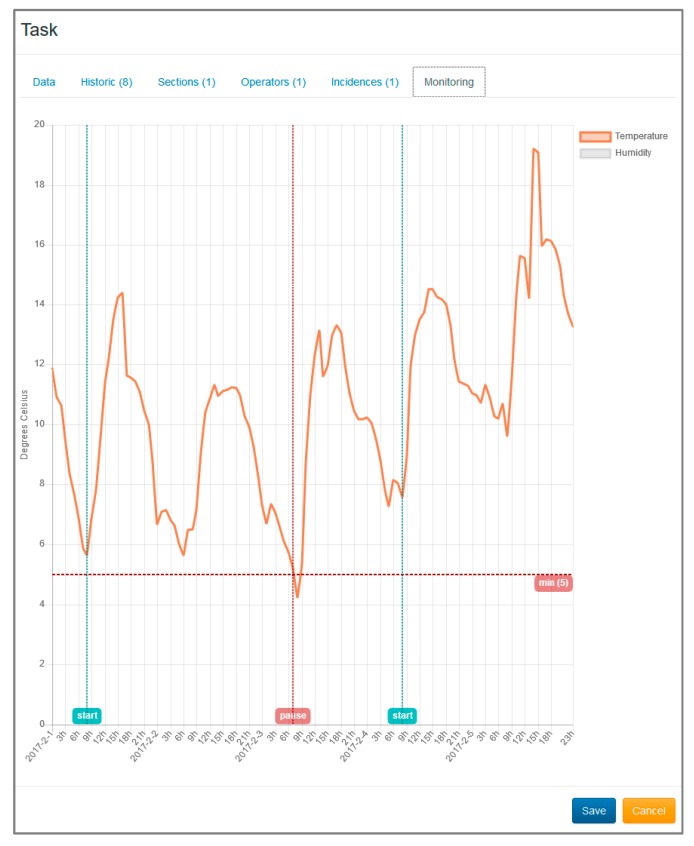
Screenshot from the LPS WebApp with the result of monitoring process.

This experiment allowed validating the integration and the joint work of all elements of the system. In this experiment, a complete cycle was completed within the construction process, consisting of the following actions:

1. Planning, through the web interface of LPS all the tasks associated with the construction of a real flat roof. In the planning, both its date of completion, as operators and sections were assigned to each task (Figure 17).
2. The relevant monitoring rules were configured for the correct execution of each task (maximum and minimum temperatures, wind, humidity …).
3. Throughout the entire implementation process, the status of all tasks and the evolution of the variables related to each one could be consulted at all times.
4. During the placement of regulation layer in the formation of slopes it was obtained a temperature below 5 ° Celsius. This resulted in the automatic creation of an incident, the pausing of the task, and the notification to the operators, foremen and site manager of the construction project (by means of a smartwatch), (Figure 18). This allowed validating the proactivity of the system. The next day, once the temperature had risen, the task was resumed.
5. Once the work was completed, it was possible to access information related to each task, including the evolution of all the variables involved and images of the moments in which specific data was taken, such as the case of slopes check. This validated the persistence of the information, which would be necessary for process debugging in case of a future problem in the roof.

The experiment was satisfactory and allowed validation of a full functional cycle of the model, from the planning to the debugging of possible errors. This revealed the adequacy of the integration of contextual monitoring as the backbone of our proposal.

## 8. Conclusions

This paper analyzes how a high percentage of the defects detected during the use phase of buildings are due to mistakes made at the construction phase of the project. These errors often occur because of the absence of control systems during the construction phase and the lack of information related to the physical variables of the environment when tasks are performed. This information determines the suitability of context to perform these tasks or on the contrary paralyze works.

This work is focused on the flat roof construction process since according to the MUSAAT Insurance Foundation, it represents the activity with the second greatest number of defects related to the above problem.

Therefore, the present work proposes a new integrated management model to improve efficiency in the construction process, specifically the construction process of flat roofs, reporting the following benefits.

- The model includes information about variables of the physical environment that affect the quality of the construction during the execution phase.
- Updated information about variables is automatically acquired and provided through intelligent sensors networks and distributed sensors offered as services.
- The proposal includes a novel sensor system specifically designed for the construction environment.
- The model integrates workers and autonomous systems into the process transparently through IoT solutions and integration techniques. This allows guiding workers through the construction tasks from the context information of the worker’s environment.
- The solution provides traceability throughout the flat roof construction process.

The proposed model is based on the use of the LPS methodology as a task management system, focused specifically on the Weekly Work Plan phase. The model includes the management of restrictions or suitability when tasks are performed, through a sensor system that acquires context information offering it to workers at all times. This information allows guiding workers along with the construction process through IoT solutions and mobile devices.

As a result, a prototype of the proposed model was made to validate the feasibility of the system in a real construction environment. This allowed us to know how the model behaves in presence of different changes of variables in a real environment, notifying at all times to all the workers involved, in an unattended and remote way, if tasks could be performed or not. In addition, information was integrated into the Kanvas system of the LPS providing site managers a global view of the state of work, updated at all times and from any place.

Finally, after the validation we concluded that information about the management of tasks and the context variables was available during the execution process carried out in the validation, and it will allow an easier identification of possible improvements of the process. As future work, an extension of this management flat roof construction process model to other phases of a construction project will be developed. Likewise, automation capabilities and traceability processes will be increased by using Business Process Management paradigm as a backbone.

## Figures and Tables

**Table 1:** Defects in roofs and in their protective layer.

General Building Defects	Roof Defects
Building in the Tiling	√
Tiled cracking	√
Lack of Adherence in Tile Parts	√
Carbonation	
Corrosion	
Efflorescence	√
Erosions	
Fissures	√
Deflections	
Dents	√
Cracks	√
Helicity	√
Dampness in construction	√
Humidity by filtration	√
Humidity by condensation	√
Subsidence	
Chemical Injuries	√
Stains	√
Living organisms	√
Punching	√

**Table 2:** Classification of the variables defined in Figure 4.

Process	Task	Variable						
		Temperature	Rain	Humidity	Wind	Slope	Presence of dust and dirt	Lack of Flatness
Previous Checks	Check base surface						S	S
	Check singular points						S	S
Layout	Lay out slope							
Slopes	Masters for joints, roof valleys and hip lines				C		S	
	Laying of the clay	C	C	C	C	S	S	
	Extended regulation layer	C	C	C	C	S	S	
	Check cement render	C	C	C	C	S	S	
Waterproofing	Priming the base with asphaltic emulsion	C	C		C	S	S	
	Resolve singular points	C	C		C			
	Placing bitumen sheets	C	C	C	C	S		
	Priming with bitumen emulsion	C	C		C	S		
	Placement of bands and pieces of adherence	C	C		C		S	
	Adhesion of the membrane to layer of slope formation	C	C	C	C	S	S	
	Placement the membrane at singular points	C	C		C		S	
Insulation	Placement of thermal insulation panels		C	C	C			
	Placement of panels at singular points		C	C	C			
Separation Layer	Placement of geotextiles	C	C	C	C			
Regularization	Make mortar screed	C	C		C	S		
Protective Layer	Floor Layout, joints and singular points		C		C	S	S	
	Tilework	C	C	C	C	S	S	
	Skirting board placement	C	C		C		S	
	Sealing points	C	C		C		S	
	Grouting	C	C		C	S	S	
	Placement of gravel	C	C		C	S	S	

**Table 3:** Sensorized variables and acceptable intervals in the works.

Environmental Conditions	Limits
Temperature	Between 5 °C and 30 °C
Rain	No rain
Humidity	<3%
Wind	<50 km/h
Work Conditions	Limits
Slope	Between 1% and 5%
Presence of dust and dirt	0
Lack of Flatness	<3 mm deviation in 2 m

**Table 4:** Hardware Device Specs.

	DS	NSN	GSN
Platform	Raspberry pi 3 Model B	Raspberry pi 1 Model B+	Raspberry pi 3 Model B
Power	Owlotech PowerBank 10,000 mAh	PowerBank Solar 15,000 mAh Levin	Wired
Storage	Samsung Micro SDHC EVO 8 GB Clase 10		
Communication (Sensor Network)		XBee Pro XBP24-AWI-001 de 2.4 GHz	
Temperature and humidity		DHT11	
Moisture (surface)	Hygrometer FC-28		
Rain		Raindrop Sensor YL-83	
Atmospheric pressure		Barometer BMP180	
Presence		Passive Infrared Sensor HC-SR501	
Tilt	Accelerometer/Gyroscope MPU-6050		
Dust	Sharp GPY2Y1010AU0F		
Image		Raspberry PI camera V2	
Wind		Photointerruptor CNZ1120	
Position	GPS Ublox NEO6MV2		
Output (user)	LCD Display 1602 HD44780		
Input (user)	Rotatory Encoder 318-ENC130175F-12PS		

**Table 5:** List of the software components and versions used.

	Name	Version
Operating System	Archlinux ARM	ARM Pi
Programming Platform	Python	3.6.0
GPIO	RPi.GPIO	0.6.3
XBee	XBee	2.2.3
erial communication	pyserial	3.2.1
I2C protocol	smbus	1.1
DHT Sensor	Adafruit-DHT	1.3.2
Camera	picamera	1.10
Image to Bytes	Pillow	4.0.0
SPI protocol	spidev	3.2
SQLite	sqlite3	3.16.2
REST Services	Flask	0.12
REST client	Request	2.13.0
